# Pathogenesis of miR-155 on nonmodifiable and modifiable risk factors in Alzheimer’s disease

**DOI:** 10.1186/s13195-023-01264-z

**Published:** 2023-07-14

**Authors:** Jia-jia Liu, Yun-fan Long, Peng Xu, Hai-dong Guo, Guo-hong Cui

**Affiliations:** 1 Academy of Integrative Medicine, Shanghai University of Traditional Chinese Medicine, Shanghai, 201203 China; 2 Department of Anatomy, School of Basic Medicine, Shanghai University of Traditional Chinese Medicine, Shanghai, 201203 China; 3 Department of Neurology, Shanghai No. 9 People’s Hospital, Shanghai Jiaotong University School of Medicine, Shanghai, 200011 China; 4 Affiliated Hospital of Jining Medical University, Jining, 272000 Shandong China

**Keywords:** Alzheimer’s disease, miR-155, Biomarker, Pathogenesis, Neuroprotection

## Abstract

Alzheimer’s disease (AD) is a common age-related neurodegenerative disease in the central nervous system and is the primary cause of dementia. It is clinically characterized by the memory impairment, aphasia, apraxia, agnosia, visuospatial and executive dysfunction, behavioral changes, and so on. Incidence of this disease was bound up with age, genetic factors, cardiovascular and cerebrovascular dysfunction, and other basic diseases, but the exact etiology has not been clarified. MicroRNAs (miRNAs) are small endogenous non-coding RNAs that were involved in the regulation of post-transcriptional gene expression. miRNAs have been extensively studied as noninvasive potential biomarkers for disease due to their relative stability in bodily fluids. In addition, they play a significant role in the physiological and pathological processes of various neurological disorders, including stroke, AD, and Parkinson’s disease. MiR-155, as an important pro-inflammatory mediator of neuroinflammation, was reported to participate in the progression of β-amyloid peptide and tau via regulating immunity and inflammation. In this review, we put emphasis on the effects of miR-155 on AD and explore the underlying biological mechanisms which could provide a novel approach for diagnosis and treatment of AD.

## Introduction

Alzheimer’s disease is a progressive neurodegenerative disease and its prevalence has been increasing with stepping into the rank of aging population [1]. An epidemiology study showed that around 50 million individuals suffer from AD worldwide at present and it is inferred to triple by 2050 [2]. It is undoubtedly that the increase of morbidity will further contribute to personal, social, and economic burdens. The pathological features of AD are extracellular amyloid-β (Aβ) deposition, intracellular neurofibrillary tangles (NFTs), gliosis, inflammation, and blood–brain barrier (BBB) disruptions, which lead to the following synaptic dysfunction, neuronal loss, and correlative clinical symptoms [3, 4]. Indeed, it is hard to make a definite diagnosis at early stages of this disease. Later neuropsychological testing, neuroimaging (MRI^18^ ,fluorodeoxyglucose-PET, and amyloid-PET) combined with biomarkers are mainly diagnostic method currently [5]. Besides, only six drugs are approved by Food and Drug Administration and put into clinical apply by 2022. They are donepezil, rivastigmine, galantamine, memantine, Namzaric®, and aducanumab [1]. The first five drugs highlight symptomatic treatment, because they merely alleviate the cognitive and behavioral deficits and improve the quality of life of the patients without blocking progression of AD fundamentally [6, 7]. Hence, there is an urgent need for exploring its underlying mechanism, reliable diagnosis, and viable medical support. miRNAs are a class of single-stranded non-coding RNA molecules with ~ 22 nucleotides in length encoded by endogenous genes. They participate in the regulation of gene expression post-transcriptionally by binding with the 3′-untranslated region of target messenger RNA (mRNA) [8]. A single miRNA can target hundreds of mRNAs due to the imperfect complementarity needed for binding and influence the expression of those genes subsequently. In general, some dysfunctional downstream targets are involved in a functional interacting pathway throughout the disease's progression. A number of miRNAs were measured and disordered in the brain, blood, or cerebrospinal fluid (CSF) of mammals when the central nervous system (CNS) suffers from lesion [9]. Hence, miRNAs act as important regulators for many neurodegenerative diseases, including AD. Recent research indicates that the serums of 7-month-old APP/PS1 transgenic mice and AD patients at different clinical stages appear to lower levels of miR-590-5p. And miR-590-5p mimics can rescue cell viability and decrease apoptosis of HT-22 and BV-2 cells under the intervention of Aβ1-42 [10]. Wang et al. observed that miR-124 levels were markedly elevated in the hippocampus, both AD patients and animal models. PTPN1, as a downstream of miR-124, can regulate synaptic plasticity via dephosphorylation of GluA2 at Tyr876 Site, suggesting synaptic function recovery mediated by miR-124/PTPN1 pathway make a valuable contribution towards AD [11]. Another study found that miR-124 could directly suppress the expression of BACE1, which plays a critical role in Aβ accumulation or the pathogenesis of AD [12]. Moreover, an RNA sequencing analysis concerning the cerebral cortex revealed that a total of 68 differentially expressed miRNAs were found between APP/PS1 mice and age-matched wild-type mice at 1, 3, 6, and 9 months of age. But the number of miRNAs which were significantly dysregulated and consistent in two age categories is quite little [13]. This result of overlapping illustrated that the expression of miRNAs will change dynamically and the aberrant miRNAs are specific during the pathogenesis of AD in the hope of serving as therapeutic targets or sensitive biomarkers.

MiR-155 is processed from the B cell integration cluster transcript, which is transcribed from the MIR155HG (miR-155 host gene) locus on chromosome 21 [14]. MiR-155 is originally most widely studied as an oncogene in a variety of neoplastic diseases, including breast cancer, hematological malignancies, and pancreatic neoplasia [15, 16]. In recent years, there is growing evidence that miR-155 is a vital regulatory factor responsible for immune response and inflammation in the development and progression of neurological disorders [17]. A systematic bioinformatics analysis was conducted and found that 13 miRNAs are involved in the pathological progress of AD, comprising miR-155 [18]. The results of in situ hybridization revealed that miR-155 was upregulated remarkably in the brain slices of human AD subjects or dementia patients caused by Down syndrome [19–21]. These results seem to be in accord with earlier research. Given lower levels of miR-155 were measured in HT-22 cells after the intervention of Aβ1–42 oligomers, miR-155 was used as one of AD-related markers and designed as fluorescent nanoparticle for diagnosing AD via binding to the probe molecule [22].

In this article, we pay more attention to the effect of miR-155 on AD and explore its underlying mechanism with the aim of providing a novel perspective for miRNA therapeutic strategies and specific biomarkers of AD.

### Potential biomarker of circulating miR-155 in AD

Currently, the concentration of 42-amino-acid isoform of β-amyloid (Aβ42) and P-tau in the CSF is considered to be the most reliable and specific biomarkers for the clinical diagnosis of AD [23, 24]. However, once the report shows a decline in the level of CSF-Aβ42 or a raised level of CSF-P-tau, it means that irreversible neurological damage, more severe pathological changes, and symptoms have taken place on account of the negatively correlation between CSF-Aβ42 and amyloid PET status [25, 26]. As well as this, CSF samples are collected by invasive lumbar puncture. Thus, it is imperative to seek a novel, safe, specific, and accurate strategy for the pre-clinical diagnosis of AD. Blood may seem like a good choice as it is easier to collect than CSF. Plasma Aβ42/40 also is recognized as a biomarker which can reflect amyloidosis in the brain accurately, especially after the improvement of inspection methods (i.e., an immunoprecipitation mass spectrometry selected reaction monitoring method, Simoa assays), yet the methods are excessively fussy [27, 28]. miRNAs, derived from the blood, are expected to be potential biomarkers for disease assessment. A previous study had reported that four differential expressed serum miRNAs, including miR-31, miR-93, miR-143, and miR-146a, were identified in AD patients via Solexa sequencing and RT-qPCR and could be taken as the non-invasive indicators for the diagnosis of AD [29]. Research on the profiling of blood plasma microRNAs of hTau mice showed that miR-155 levels were dramatically decreased at 16 weeks old when early tau aggregation rather than clinical symptoms was showed. And receiver operating characteristic curve analysis showed that miR-155 could accurately distinguish pre-symptomatic tauopathy from control (AUC = 0.95). This discovery will be beneficial for early clinical diagnosis of tauopathy, such as AD [30]. Guedes et al. found that the expression of miR-155 in blood-derived monocytes varied considerably between AD patients and mild cognitive impairment (MCI) patients/healthy controls. However, no difference of miR-155 was observed between healthy controls and MCI patients which implied that miR-155 has preferable sensitivity and specificity to diagnose AD [31]. Given that miRNAs are susceptible to being degraded by ribonuclease, several miRNAs could achieve rather steady structure via embedding themselves in the exosomes or binding to high-density lipoproteins in peripheral circulating blood [32–34]. Li et al. demonstrated that intraperitoneally administered lipopolysaccharide (LPS) can increase the expression of miR-155 in serum-derived exosomes and induce the activation of microglia and astrocytes, contributing to severe neuroinflammation [35]. In other words, serum exosomal miR-155, as a mediator, is involved in the progression of neuroinflammation or indicates the occurrence of some diseases. Another study discovered a trend towards descending levels of serum exosomal miR-155 in dementia patients, but no significance. It does not mean that serum exosomal miR-155 cannot serve as an early diagnostic indicator [36]. Because those dementia patients consisted of vascular dementia patients and AD patients with or without medication. This controversial conclusion may be interfered with unclear classification or limited sample size.

With reference to published documents, a thought-provoking question was raised. Namely, not only are there few repots about blood-derived miRNAs biomarkers in AD, but there were several inconsistencies in the results of those surveys [37]. To avoid this phenomenon, the clinical dementia stages, the origin of miRNAs, patient samples, and detection methods will need to be further classified, identified, enlarged, and standardized. After overcoming those factors, circulating miR-155 might be a valuable biomarker for the diagnosis of AD.

### Neuropathogenesis of miR-155 in AD

#### MiR-155 contributes to Aβ pathology

It is well known that amyloid cascade hypothesis is a predominant theory in the etiopathogenesis of AD. This hypothesis proposed that progressive aggregation of Aβ peptides could precipitate the formation of soluble oligomers, insoluble fibrils, and senile plaques and therefore trigger a series of cascade reaction, including synaptotoxic, inflammatory responses, oxidative injury, neuronal degeneration, and tau hyperphosphorylation [38]. The generation of Aβ is closely related with classical “amyloidogenic pathway.” Briefly speaking, amyloid precursor protein (APP) undergoes sequential cleavages by β-secretase and γ-secretase and then generates various Aβ fragments [39]. Dynamic equilibrium of Aβ production can be maintained under physiologic conditions but not the pathologic condition of AD. Thus, either excessive generation of Aβ or insufficient clearance of Aβ can accelerate the pathological process of AD. Research showed that disordered miRNAs are closely associated with disease pathology and they take part in Aβ production via medicating the amyloidogenic pathway or nonamyloidogenic pathway. For instance, miR-30a-5p, miR-140-5p, and miR-221 could inhibit their common downstream target ADAM10 and led to the subsequent decrease of neuroprotective sAPPα [40–42]. ADAM10, as the leading member of α-secretase, is responsible for nonamyloidogenic pathway by competitive cleaving APP. MiR-29c-3p, miR-124, or miR-15b repressed BACE1 expression and aggravate AD from the amyloidogenic point of view [12, 43, 44].

**Fig. 1:**
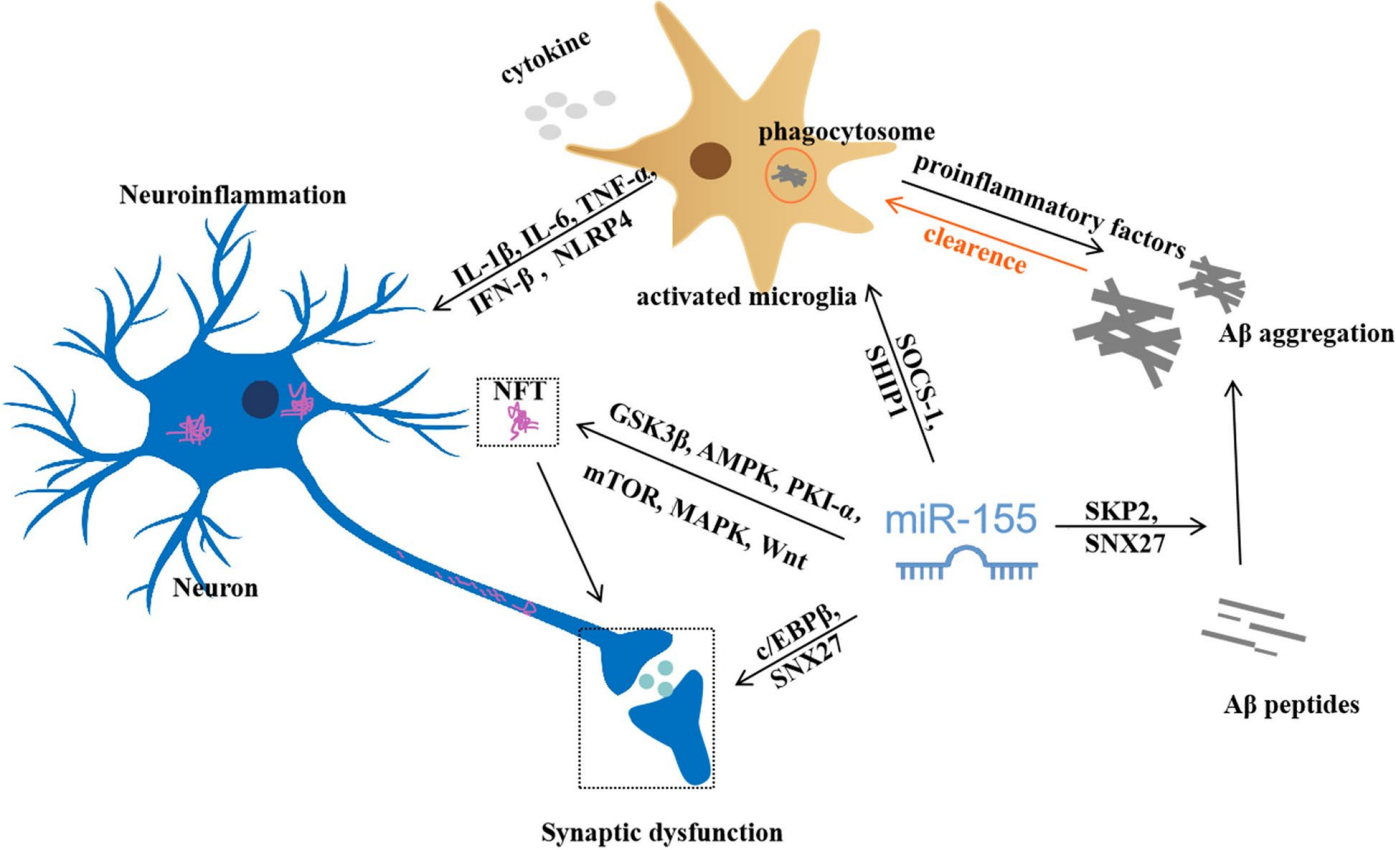
Neuropathogenesis of miR-155 in AD. miR-155 regulates the deposition of Aβ by inducing SKP2 or SNX27. miR-155 might be involved in NFT by regulating GSK3β, AMPK, PKI-α, mTOR, MAPK, or Wnt pathways, which evolves from assembled hyperphosphorylated tau protein and induces synaptic dysfunction. miR-155 was also involved in synaptic dysfunction by regulating c/EBPβ/SNX27 signaling pathway. miR-155 can activate microglia via targeting SOCS-1 or SHIP1. The activated microglia can phagocytize aggregated Aβ but also can release pro-inflammatory cytokine, which accelerates Aβ aggregation and leads to neuroinflammation

The effects of miR-155 on Aβ pathology are summarized in Fig. 1. Wang et al. demonstrated that the expression of miR-155 was upregulated significantly in the hippocampus of APP/PS1 mice. And knockdown of miR-155 could partially reverse Aβ deposition in encephalic parenchyma and regulate the expression of Aβ generation-related proteins in N2a cells via targeting SKP2 [45]. Human SH-SY5Y cells, overexpressed APP with the 695-amino-acid Swedish mutation (APP695), could enhance the levels of the real toxic Aβ trimers/tetramers species (~ 20 kDa) and miR-155 dramatically after differentiation induced by RA [46]. Presenilin (PSEN 1), as the catalytic part of the γ-secretase complex, can indirectly regulate Aβ level [47]. In this article, another AD cell model, the neurons differentiated from iPSC which generated from an AD patient carrying PSEN1ΔE9 mutation, was also performed and found that it could increase the levels of larger oligomers and decrease the expression of miR-155 significantly. Though authors did not make further efforts to confirm the effect of miR-155 on APP gene expression or Aβ oligomerization, differentially expressed miR-155 in both SH-SY5Y cells and iPSC-derived neurons implied its potential for Aβ pathology. The inconsistent alterations of miR-155 might be dependent on specific AD models. In addition, Aβ accumulation, as a shared feature, is widely reported to be the major contributor to drusen deposits in age-related macular degeneration (AMD) patients [48]. Intravitreal injection of Aβ oligomer solution is commonly used to imitate AMD in vivo. A previous study has shown that elevated miR-155 levels were found in the retina of rats receiving intravitreal injection of Aβ oligomers [49]. Then, as an important regulatory factor in inflammation, miR-155 may trigger the following retinal inflammation. But the Aβ oligomer was directly responsible for the dysregulated miR-155. A recent study proved that the expression of miR-155 extracted from the retina was upregulated significantly in the 3xTg-AD mice at different ages, whereas abundant Aβ deposition was measured in the retina of AD mice, especially in the RPE retinal layers [50]. It is undeniable that there is a cause-and-effect relationship between Aβ pathology and miR-155. A miRNA seq analysis revealed that miR-155 is truly upregulated in the hippocampus of 10-month-old APPtg mice. And 28 clinically diagnosed AD patients showed consistent results. The levels of soluble and insoluble Aβ40 and Aβ42 were obviously raised in the hippocampus of the amyloidosis mouse model and high correlated with the miR-155 levels via Spearman correlation and FDR* p*-value adjustment [51]. And their predicted targets of miR-155 were reported to be involved in Aβ generation or tau kinases (e.g., GSK3β). We guess that there is a complex crosslinking reaction between Aβ metabolization and miR-155 and the precise mechanisms need to be further investigated. Noteworthy, enhancing the expression of miR-155 did not influence cognitive function through intracerebroventricular injections in wild-type mice, suggesting miR-155 is involved in the genotype of AD, particularly in APP mutation.

#### MiR-155 contributes to Tau pathology

It is generally accepted that NFTs are another pathological hallmark of AD. NFTs evolve from paired helical filaments which are assembled by hyperphosphorylated tau protein [52]. Tau, also known as microtubule-associated protein tau, is mostly expressed in the axons of neurons or ocular neurons and involved in microtubule assembly, stabilization, and axonal transport [53]. As a “natively unfolded” protein, tau protein will misfold and accumulate abnormally due to improper post-translational modifications and genetic mutations, leading to neurodegenerative disorders ultimately [54]. Anomalous post-translational modifications comprise phosphorylation, acetylation, glycation, ubiquitination, and truncation [55]. Among them, phosphorylation has been the most extensively studied. Tau is hyperphosphorylated by activated kinases in its C-terminus and then leaves the cytoskeleton, resulting in disrupt stabilization, synaptic dysfunction, and increased aggregation effects eventually [56].

In order to figure out whether deregulated miRNAs in AD brain were regulated by tau pathology, hTau transgenic mice were applied in this experiment. The hTau mice which express all six human tau isoforms are exceedingly similar to the tau pathology in AD patients and display hyperphosphorylated tau protein in neurons at 3 months old [57, 58]. Ryan et al. discovered that plasma miR-155 was downregulated significantly at 16 weeks old and maintained lower levels till 53 weeks old in hTau mice, indicating miR-155 is involved in both pre-symptomatic primary tauopathy and symptomatic tauopathy. And the bioinformatic analysis revealed that miR-155 might regulate neuropathological lesions induced by tau via four signaling pathways, including mTOR, MAPK, Wnt, and T-cell receptor [30]. In addition, massive accumulation of phosphorylated Tau (P-tau) and upregulated miR-155 were identified in the retina of 3xTg-AD mice at 15 months old. Tau aggregation caused by conformational changes or hyperphosphorylation is reported to be found in the hippocampus of 3xTg-AD mice in the 12th month [50]. This animal model is universally accepted and the familial Alzheimer’s disease of human being approaches most. Retinal P-tau might be an alternative method to detect early pathological changes in AD owing to the pathophysiological homology between the retina and the brain [59]. Hence, miR-155 may be bound up with tauopathy. Surprisingly, most of cells accompanied with hyperphosphorylated tau protein also expressed high levels of miR-155 in the brain tissues of Down’s syndrome dementia [21]. Though the mechanisms of AD and Down’s syndrome dementia are different, both of them can cause abundant hyperphosphorylated tau protein aggregation in the brain. And Down’s syndrome can increase the risk of AD. This study further strengthened the evidence linking miR-155 with P-tau. On the other hand, miR-155 could suppress the expression of C/EBPβ via directly binding with the 3′noncoding region of C/EBPβ mRNA and thereby decrease SNX27, causing synaptic dysfunction in Down syndrome mice [60]. Currently, it is demonstrated that knockout of tau gene might damage long-term potentiation (LTP), an important manifestation of synaptic plasticity [61]. While conditional knockout of miR-155 alone also can induce the impairment of LTP in wild-type mice [20]. Thus, we have reasons to believe there is a link between miR-155 and tau under complex pathological conditions on the basis of the abovementioned published documents (Fig. 1).

MiR-132 can directly target tau mRNA to regulate its expression [62]. By contrast, effectors of abnormal tau phosphorylation are widely studied. Three kinds of protein kinases which are in charge of tau phosphorylation are as follows: Proline-Directed Protein Kinase (PDPK), Non-Proline Directed Protein Kinase (Non-PDPK), and Tyrosine Protein Kinases (TPK) [63]. The most common of these are Glycogen synthase kinase-3 (GSK-3), C-Jun amino-terminal kinase (JNK), Adenosine-monophosphate activated protein kinase (AMPK), Cyclic AMP (cAMP)-dependent protein kinase (PKA), Cyclin-dependent kinase 5 (cdk5), and so on. For example, miR-219-5p or miR-23-3p alleviated tau pathology and rescued cognitive function by targeting GSK3β in AD [64, 65]. Researchers have investigated that miR-155 participates in inflammatory responses induced by LPS in acute inflammation model [66], T cell proliferation in cardiac allograft rejection [67], and epithelial-mesenchymal transition in radiation-induced pulmonary fibrosis [68] through directly targeting GSK3β. Furthermore, miR-155 also targeted AMPK [67, 69] and PKI-α [70] which can reduce nuclear PKA activity in other diseases. Those pivotal downstream targets of miR-155 may be a breakthrough to investigate tauopathy mediated by miR-155.

#### MiR-155 contributes to neuroinflammation

Apart from those two typical pathological features, neuroinflammation has generated a lot of interest. Although neuroinflammation as the cause or the outcome of AD remains unclear, a sustained inflammatory response has been observed throughout the whole pathological process of AD [71]. Microglial cells, as the resident innate immune cells in the CNS, play an essential role in regulating neuroinflammation. In the state of physiology, microglial are in a resting state and explore the surroundings via extending and retracting continually. They perform the functions of monitor, neurogenesis, neurotrophy, and synapse plasticity. Under threat, microglia can be activated quickly, migrate to the lesion through chemokine receptors, and recognize danger-associated molecular patterns (DAMPs) or pathogen-associated molecular patterns (PAMPs) through its receptors, including Toll-like receptors (TLRs), NOD-like receptors (NLRs), C-type lectin receptors (CLRs), and so on [72]. In addition, microglia release proinflammatory cytokines to recruit additional cells which is a double-edged sword in the progression of AD [73].

It is generally accepted that miR-146a, miR-125b, and miR-155 show a progressive link to the neuroinflammatory signals. MiR-146a targets C/EBP, whereas miR-125b regulates multiple inflammatory mediators [40]. It was reported that the expression of miR-155 presents an age-dependent increase in the 3xTg AD model. Compared with their WT littermates, extensive gliosis and increased IL-6 and IFN-β levels were observed in 3xTg AD mice at 12 months old, prior to senile plaque deposition, suggesting the association of neuroinflammation in the pathogenesis of AD. And the levels of miR-155, IL-6, and IFN-β were also upregulated in both microglia and astrocytes with the treatment of Aβ fibrils. This effect of miR-155 on inflammatory signaling is achieved by targeting its downstream SOCS-1 which is responsible for the upregulation of IL-6 and IFN-β [74]. Early inflammatory events may accelerate the formation of Aβ deposition; Aβ deposition conversely increases the production of inflammatory factor, which lead to a continuing viscous cycle. MiR-155 could play an irreplaceable role in this process. Furthermore, the microarray expression profiling analysis of miRNA confirmed that miR-155 was the most significantly upregulated miRNA when primary microglia were stimulated by LPS. And this result was verified by q-PCR [75]. It indicates that miR-155 plays a crucial role in the inflammatory response mediated by activated microglia. In view of the significance of microglia, Aloi et al. observed microglial response to fibrillar Aβ1-42 under the stimulation of LPS. Preliminary results showed that the expression of miR-155 rapidly raised on the 1st day and declined slightly latter, and still maintained at a high level on the 3rd day. They found that the absence of miR-155 could alter the ability of microglia to transfer internalized Aβ to lysosomal compartments [76]. In another study, a rat model of AD was established by LPS in the pattern of intraperitoneal injection similarly. The results showed that significant increases in Aβ1-42, p-tau, miR-155, TNF-α, and p-AKT levels were observed after the intervention of LPS. However, the levels of IL-10 and SHIP-1 exhibited an opposite trend [77]. The analysis of Pearson’s correlation coefficient revealed that there exists a positive correlation between miR-155 levels and p-AKT/TNF-α. It is confirmed that SHIP-1 is a downstream of miR-155 and a negative regulator of p-AKT [78]. Taken together, increased miR-155 is involved in neuroinflammation through targeting SHIP-1/p-AKT axis. Interestingly, after intraventricular injection of miR-155 inhibitor, the upregulated IL-1β, IL-6, and TNF-α could be reversed partly in AD rats [79]. This result further confirmed the close relationship between miR-155 and proinflammatory cytokines. To sum up, miR-155 is a promising target to control neuroinflammation in AD (Fig. 1).

### Pathogenesis of miR-155 in AD-related modifiable risk factors

**Fig. 2:**
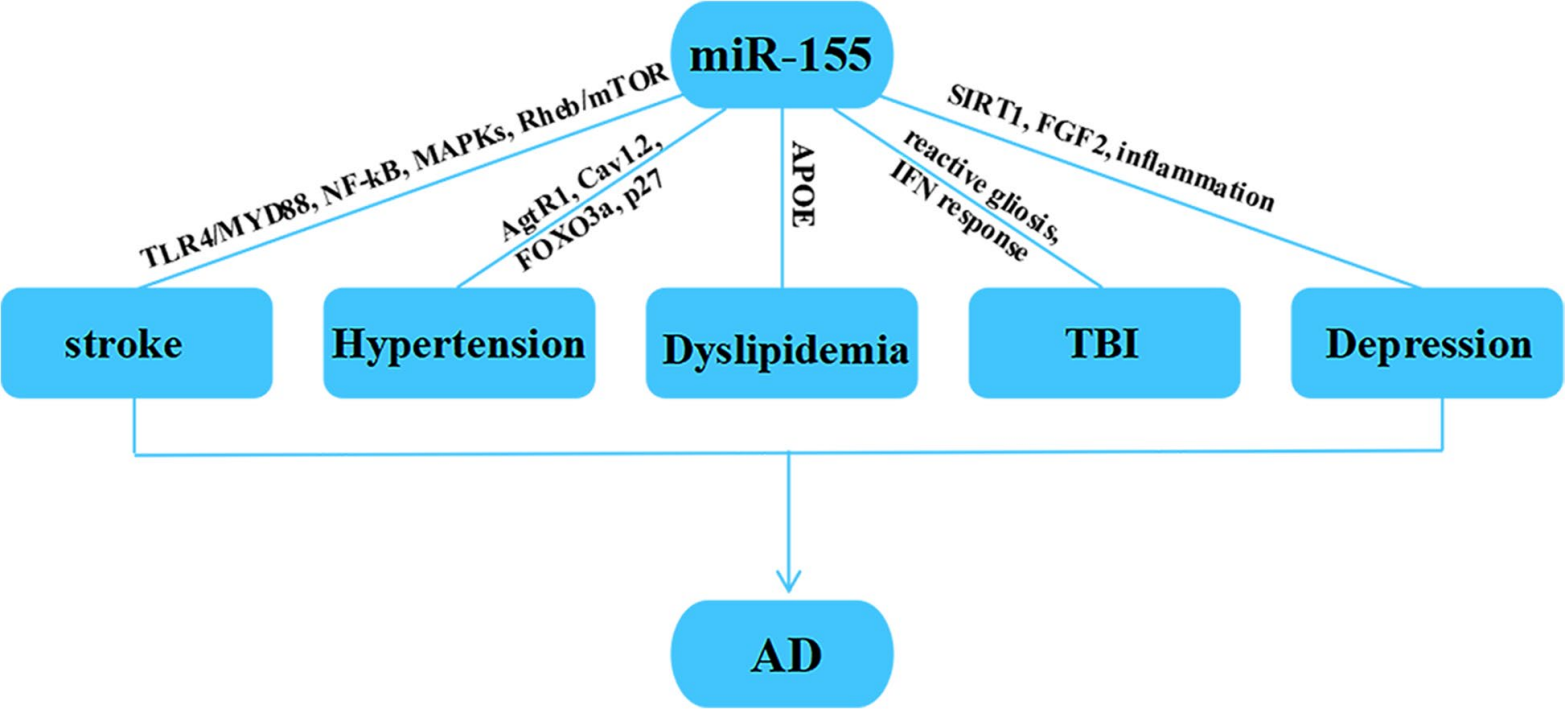
Pathogenesis of miR-155 in AD-related modifiable risk factors. Hypertension, stroke, TBI, dyslipidemia, and depression are common AD-related modifiable risk factors. miR-155 regulates hypertension by inducing AgtR1, Cav1.2, FOXO3a, or p27. miR-155 might be involved in cerebrovascular diseases, including stroke and TBI. Among that, miR-155 is involved in stroke by regulating TLR4/MYD88, NF-kB, MAPKs, or Rheb/mTOR pathways, but regulate TBI on the basis of reactive gliosis, IFN response. miR-155 is involved in dyslipidemia by regulating APOE, especially in atherosclerosis. miR-155 can aggravate depression via targeting SIRT1, FGF2 or inducing inflammation

Although age acts as the greatest nonmodifiable risk factor to trigger AD, numerous modifiable risk factors have been confirmed to increase the odds of AD, which accounts for approximately 35% of the total risk of AD [80, 81]. Now studies concerning modifiable risk factors are primarily focused on hypertension, cerebrovascular diseases, dyslipidemia, depression, diabetes, obesity, physical inactivity, and smoking [82]. This discovery provides evidence for a downtrend in age-adjusted AD incidence and prevalence in high-income countries [83]. Thus, it is particularly important to make full understanding on those modifiable risk factors and achieve early management. Here we will briefly introduce the role of miR-155 in certain pivotal modifiable risk factors of AD (Fig. 2).

#### MiR-155 contributes to hypertension

Several clinical studies have provided evidence to support that hypertension, especially midlife hypertension, increased the risk of AD [84–86]. Furthermore, in mouse models, extensive Aβ deposition and the broken BBB were observed in the hypertension model induced by transverse aortic coarctation or chronic Angiotensin II infusion [87]. As people get older, the prevalence of hypertension increases significantly. DuPont et al. discovered a huge decline in the expression of miR-155 in aortas with aging consist with previous investigation in peripheral blood mononuclear cells [88]. They authenticated that miR-155 is responsible for relieving vasoconstriction in the aging vasculature via targeting the L-type calcium channel (LTCC) subunit Cav1.2 and the angiotensin type-1 receptor (AgtR1) [89]. The expression of miR-155 was found to be downregulated in the aorta of 16-week-old spontaneously hypertensive rats compared with age-matched Wistar-Kyoto rats. And Pearson and Spearman correlation analysis revealed a strongly negative correlation between blood pressure and miR-155 [90]. In addition, researchers further demonstrated that the mechanisms of miR-155 involved in regulating the blood pressure might be related to the direct repression of AgtR1 at the translational level which mediates Angiotensin II and therefore regulates vasoconstriction [91]. In contrast, other studies had found that inhibition of miR-155 can significantly depress blood pressure which were mainly based on endothelial cells and vascular smooth muscle cell dysfunction. The possible mechanisms are the interactions between miR-155 and its targeting molecules, including FOXO3a, P27, eNOS, and NPPA [92–94]. Among those dysregulated miRNAs, miR-214 also medicated pathological changes of hypertension via targeting eNOS [95]. Pankaj Arora et al. had identified that miR-425 can also regulate the production of ANP through silencing NPPA mRNA in an allele-specific manner [96]. Interestingly, the combination of miR-155 and miR-425 could reach an even better performance in terms of lowering blood pressure, than either miRNA alone. The synergism or equal efficacy of different miRNAs may be partially attributed to their common targets. Additionally, miR-105 is involved in regulating blood pressure in the same way [97]. All of these suggested that miR-155 play an essential role in hypertension caused by aging. It is worth mentioning that miR-155 expression patterns are opposite in the brain or blood vessel with aging. Nevertheless, both dysregulated miR-155 can aggravate the proceeding of AD, directly or indirectly. When we attempt to find a therapy for AD from the perspective of hypertension, enhancing miR-155 might be a good choice according to the above reports. However, this improving strategy must be target smooth muscle cells specifically, instead of other cell types (e.g., endothelial cells, neuron, or microglia), in order to avoid exacerbating AD pathology. Thus, we cannot simply the only concern of specific single events under complex pathological conditions. It is necessary to figure out expression patterns, functional patterns, and crosstalk of miR-155 in different systems throughout the whole disease.

#### MiR-155 contributes to dyslipidemia

Dyslipidemias present the abnormal metabolism of lipoproteins which mainly include total cholesterol, low-density lipoprotein cholesterol, triglycerides, and high-density lipoprotein cholesterol [98]. Upregulated miR-155 can prevent β-to-α-cell reprogramming by supressing MafB and improves the adaptation of β-cells to insulin resistance in hyperlipidemic mice [99]. Given the brain is rich in cholesterol, higher risk of AD may be attributed to elevated cholesterol levels [82]. There have been a couple of studies suggesting elevated cholesterol levels in the peripheral circulation compromise the integrity of BBB and elevated cholesterol levels in the central nervous system impair synaptic plasticity and induce tau hyperphosphorylation [100, 101]. These findings further verify the above inference. APOE, as the most abundant apolipoprotein in the CNS, is responsible for cholesterol transport from the astroglia to the neuronal compartment [102]. The absence of APOE could aggravate the aggregation of Aβ significantly. Among those isoforms of APOE, the ε4 allele is closely related not only with a higher risk of atherosclerosis, but also higher risk of late-onset AD [103]. Although the direct effect of miR-155 on the APOE mutation-mediated AD has not been reported, numerous reports reveal the important role of miR-155 in dyslipidemia. APOE − / − mice were employed to establish experimental atherosclerosis model via feeding high cholesterol food. Elevated miR-155 levels were observed remarkably in the aortic tissue and reported to aggravate the lesion in APOE − / − mice by regulating SOCS1/STAT3/PDCD4 axis or MEK/ERK/NF-κB pathway or NoxA1-p47phox complex signaling pathway [104–106]. Namely, miR-155 may be involved in dyslipidemia via affecting numerous coding transcripts. In addition, Teter et al. examined the expression of miR-155 in APOE3-HU mice, with or without the transgene of 5 FAD mutations in the human APP and PS1 genes. The results showed that the expression of miR-155 was significantly upregulated in the cortex of APOE3 + 5xFAD mice in comparison with littermates [107]. These data are not very meaningful because of multiply variables. But that will provide a method for profound study on the interaction between miR-155 and APOE in AD.

#### MiR-155 contributes to cerebrovascular diseases

It is generally believed that cerebrovascular diseases are connected with AD closely. Autopsy reports showed that most patients who had been diagnosed with AD suffered from Aβ amyloid angiopathy and arteriolosclerotic small vessel disease [108]. Given cerebral blood flow reduction is the most notable impact of cerebrovascular diseases, they might be involved in Aβ production and Aβ clearance by vascular mediated systems, especially BBB [109]. There are numerous reports about the effect of miR-155 on cerebrovascular diseases, including traumatic brain injury, ischemic stroke, and cerebral hemorrhage. Controlled cortical impact could increase the expression of miR-155 in the injured cortex region even in microglia/macrophages isolated from the injured cortex in the model of experimental traumatic brain injury (TBI). Intracerebroventricular administration of miR-155 antagomir attenuated the elevated expression of miR-155 and pro-inflammatory cytokine induced by TBI in the regions of injured cortex and hippocampus and accelerated the recovery of cognitive function [110]. Unexpectedly, Harrison et al. found that miR-155 knock-out mice displayed more reactive gliosis, increased neurodegeneration, and a decline in the IFN response after TBI. Fluorescence in situ hybridization revealed that miR-155 was mainly located in cell nucleus. This indicated that miR-155 may participate in neuronal injury by mediating gene expression changes in non-canonical ways [111]. Another found that miR-155, as a mitochondria-associated miRNA, was involved in mitochondrial dysregulation by suppressing the expression of PGC-1 in the striatum at all time points [112]. Similarly, the increased miR-21 may cause persistent oxidative stress and damage mtDNA via targeting SOD2 at the early stages of TBI. However, different phenomena were discovered that the level of miR-155 was significantly elevated in both cytoplasm and mitochondria at 1 day following injury and then returned to baseline levels at 7 days post-injury when researchers turned to hippocampus [113, 114]. These reflect tissue specificity of miRNAs expression in the progression of the disease. Overall, miR-155 has an influence on the pathophysiology of TBI, but its exact mechanism remains to be addressed. In addition, ischemic stroke also occupies an important position. miR-155 injured the microvascular tight junctions via suppressing its downstream ras homolog enriched in brain (Rheb) and thus broke the integrity of BBB, aggravated brain edema, and induced delayed neuronal death ultimately in the pathological process of cerebral ischemia–reperfusion injury [115]. TLR4/MYD88, IRF2BP2, MafB, DUSP14, and Rheb/mTOR signaling pathways were also reported to be involved in the miR-155-mediated nerve injury after stroke [116–119]. To sum up, miR-155 participated in the pathological mechanisms of cerebral ischemia stroke by regulating inflammation or cell apoptosis pathway due to distinct targets. It is worth emphasizing that miR-155/MafB axis also contributed to dyslipidemia and atherosclerosis which is one of AD-related modifiable risk factors. Various systems may realize cross interference via miRNAs in complicated human body conditioning system, which partially accounts for the impelling action of AD-related modifiable risk factors. Throughout preclinical studies currently, miR-155 expression patterns seem to be consistent in the various cerebrovascular diseases, including AD, stroke, and TBI. Elevated miR-155 may aggravate brain injury via inhibiting down-stream genes.

#### MiR-155 contributes to depression

It is estimated that around 30% of Alzheimer’s patients simultaneously suffer from depression [120]. The presence of depression accelerates the progression of AD leading to cognitive dysfunction prematurely [121]. The mechanisms underlying the association between depression and AD remain unclear. Inflammation, serotonergic system and vascular disease may be potential mechanisms for building bridges between them [122]. A study examined the dysregulated miRNAs of 84 patients with major depressive disorder and 43 healthy controls in peripheral blood mononuclear cells. The result reflected that the level of miR-155 was downregulated significantly in peripheral blood mononuclear cells of depressive patients, but began to recover after antidepressant treatment [123]. The interaction between miR-155 and SIRT1 may be an important mediator in the pathological process of major depression disorder [124]. SIRT1, served as a potential target for treating depression, can also be suppressed by miR-124, miR-9, or miR-135b-5p, influencing depression-like behavior in mice [125–127]. Furthermore, SIRT1 exerts neuroprotective effect on cerebral ischemia by regulating inflammatory and apoptotic pathways [128]. miR-155/SIRT1 axis in cerebrovascular diseases remains to be explored in further research. Fluoxetine, a sort of selective serotonin reuptake inhibitor, is usually prescribed to treat depression. Dai et al. discovered that with the intervention of fluoxetine, the expression of miR-155 and pro-inflammatory factors descended significantly along with a raise in β-catenin protein [129]. Its pathogenesis may be related to the inhibition of the Wnt/β-catenin signaling pathway. In addition, Chao et al. demonstrated that Saikosaponin d, as an effective traditional Chinese medicine for treatment of depression, could ameliorate depression-like behaviors by regulating the miR-155/FGF2 signaling pathway [130]. And they used the dual luciferase reporter gene assay system to confirm that FGF2 is one of the target genes for miR-155 or miR-497. FGF2 is a multi-functional growth factor and plays a crucial role in the development of the CNS containing synaptic plasticity, neuronal growth, and adult neurogenesis [131, 132]. Previous study showed that bilateral hippocampal injection of AAV2/1-FGF2 could improve cognitive impairment, encourage neurogenesis, and decrease Aβ deposition in APP/PS1 mice [133]. Therefore, we proposed a hypothesis that miR-155 may be a pivotal bridge linking depression with AD via chronic inflammation and neurotrophic signals.

### Pathogenesis of miR-155 in AD-related adaptive immune

The brain is widely regarded as an immune privileged organ that is mainly attributed to the physiological properties of the BBB [134]. BBB can strictly restrict immune cell infiltrate from the peripheral circulation into the brain parenchyma and wouldn't elicit adaptive immune responses [135]. Accumulating evidences have indicated that BBB permeability increases in a manner of age-dependence and further get worse when suffering from AD [136, 137]. This provides a pathway for immune cells access to CNS, especially T lymphocytes. Consistently, the autopsy report showed that the brain parenchyma appears more cluster of differentiation (CD) 3 + T cells in most of AD patients than in control cases [138]. So far, several studies have confirmed the importance of infiltrated T cells in the development of AD.

T cells are usually divided into two subgroups the CD4 + T cells and the CD8 + T cells. CD4 + T cells are further classified into several subsets including regulatory T cells (Tregs), follicular helper T (Tfh) cells, and T helper (Th) cells (Th1, Th2, Th17, Th22) according to the distinct expression of surface molecules and endogenous production of cytokines. CD8 + T cells mainly differentiate into cytotoxic T lymphocytes (CTL). The CTLs eliminate infected target cells directly. During the pathological process of AD, CD8 + T cells could induce synaptic dysfunction and neuronal apoptosis by interacting with neurons directly. These neurons involved in this immune response are reported to express major histocompatibility complex (MHC) class I molecules, which are responsible for presenting antigens from antigen presenting cells (APC) to T cells [139]. In addition, the hippocampal RNAseq analysis performed by M.S.Unger also corroborated this claim. They found that the expression of neuronal- and synapse-related genes were dramatically upregulated in the hippocampus of APP/PS1 mice after treating with an anti-CD8 antibody [140]. However, CD4 + T cells eliminate infected target cells indirectly by interacting with other immunoreactive cells, including microglia and CTLs. Microglia can be activated by Aβ plaques and express MHC class II, CD80 and CD40, similar to the function of APC. Thus, infiltrated CD4 + T cells can be activated and carry out effector functions by interacting with microglia [141]. On the contrary, activated CD4 + T cells can affect the activation state of those microglia via secreting various cytokine. Th1 and Th17 cells are usually considered as proinflammatory T cell subtypes and product distinct cytokines IFN-γ, TNF-α and IL-2. Microglia can be activated by IFN-γ and alter phenotype, thereby contributing to Aβ deposition in AD [142]. However, Th2 cell, as an anti-inflammatory T cell subtype, secretes cytokines IL-4, IL-5, IL-10, and IL-13 and transplanted Aβ-specific Th2 cells could reverse cognitive impairment through the tail vein in APP/PS1 mice [143]. Hence, there is convincing evidence that the T cells is closely associated with the pathological responses of AD.

**Fig. 3:**
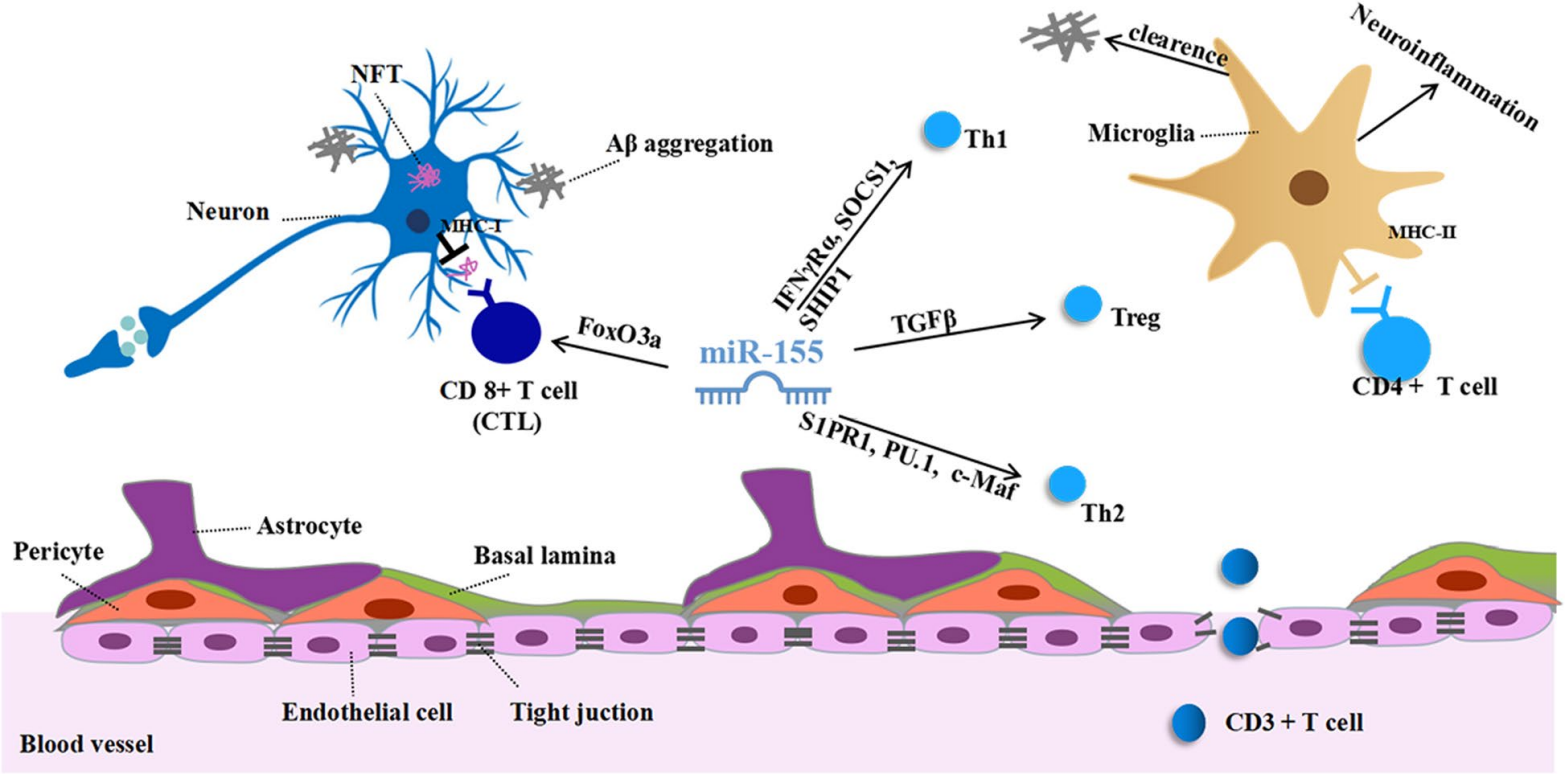
Pathogenesis of miR-155 in AD-related adaptive immune. CD3 + T cells can get access to the brain parenchyma via injured BBB. CD8 + T cells mainly differentiate into cytotoxic T lymphocytes (CTL). MiR-155 can regulate CD8 + T cells by regulating FoxO3a negatively. CD4 + T cells eliminate infected target cells indirectly by interacting with microglia. miR-155 promotes Th1 cell polarization by targeting IFNγRα, SOCS1, and SHIP1. Moreover, miR-155 can regulate the migration, differentiation, and function of Th2 cell by mediating S1PR1, PU.1, and c-Maf respectively. And miR-155 can facilitate Treg cell development via TGFβ signaling pathway. The activated microglia have different effects due to its phenotype

In terms of adaptive immune, miR-146 and miR-155 are among the first and most studied miRNAs. Some studies have found that miR-146 control Treg cells, Th1 cells and CD4 + T cells respectively through TRAF6/NF-ĸB/FoxP3 pathway, PKCε and the IFN pathway [144]. More and more researchers have already realized the importance of miR-155 in the biology of lymphocytes, especially in T cells at present. It is reported that the absence of miR-155 could significantly decrease the accumulation of CD8 + T cells in glioma. The inhibitory effects were attributed to descending proliferation and invasiveness modulated by FoxO3a/AKT/Stat5 axis. And miR-155 was recognized as a negative regulator of FoxO3a via luciferase reporter gene assay [145]. However, Cassidy et al. found that the inhibition of miR-155 has a beneficial effect in the brain during infection. Because aggregated CD8 + tissue-resident memory T cells induced by neuroinvasive listeria monocytogenes could cause post-infectious neuroinflammation, but were restrained by miR-155 [146]. Thus, the interplay between miR-155 and CD8 + T cells remains complex. Recently, Li Chen and colleagues summarized that miR-155 is involved in the activation, differentiation, function, and apoptosis of CD4 + T cell by targeting various genes in detail [147]. For example, miR-155 promotes Th1 cell polarization by targeting IFNγRα, SOCS1, and SHIP1. Moreover, miR-155 can regulate the migration, differentiation, and function of Th2 cell by mediating S1PR1, PU.1, and c-Maf respectively. And miR-155 can facilitate Treg cell development in the thymus by ensuring the proper maturation of medullary thymic epithelial cells via TGFβ signaling pathway [148]. Studies such as these are extremely common. Given the reliability of interaction between miR-155 and T cells, miR-155 might be involved in AD through T cells (Fig. 3). This finding provides a new perspective for studying the mechanism of AD and exploring novel treatment strategies. But the role of T cells is not immutable and appears to be influenced by a number of factors, including pathogenic factors, the stage of disease progression, the repertoire of inflammatory insults, and the ratio of specific immune cells types. So, the pathogenesis of miR-155 in AD-related adaptive immune needed remains a problem for further research.

## Conclusion

**Fig. 4:**
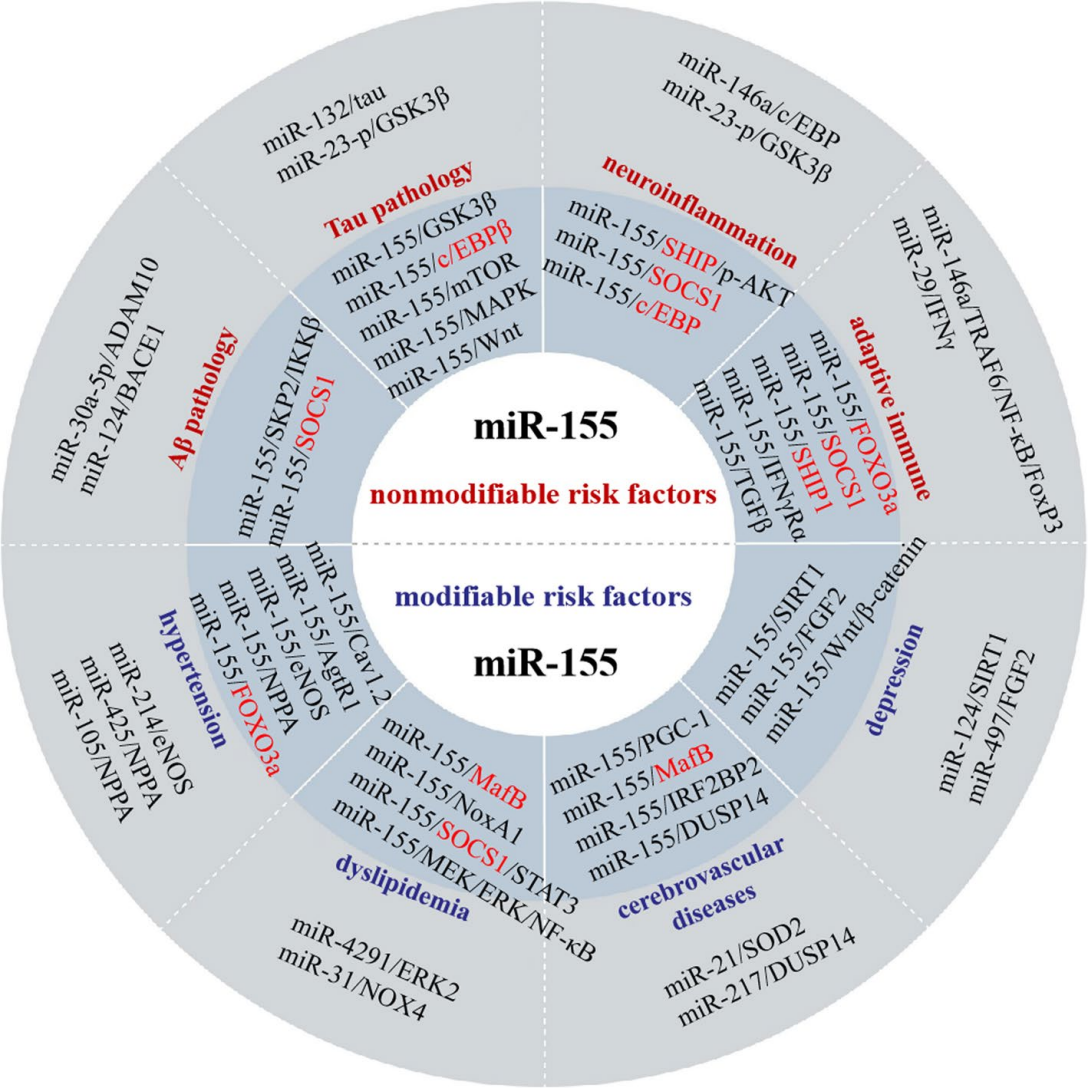
The schematic diagram summarizing the proposed mechanisms of miR-155 in AD and complex crosstalk mechanism

AD is a complicated neurodegenerative disease which is affected by multiple etiological factors. All factors are highly interactive and almost never occur in isolation from each other indeed. We have discussed that miR-155 might modulate numerous mechanisms for contributing to those etiological factors in AD, including nonmodifiable factors and modifiable factors (Table 1). In addition, miR-155 is expected to serve as a potential biomarker for the early diagnosis of AD. The present researches are focused on microglia-mediated inflammatory response. Although a high correlation between miR-155 and typical pathological hallmarks was found, there is no defined evidence to support this conclusion. As mentioned above, expression patterns and the mode of action of miR-155 in some AD-related modifiable risk factors are inconsistent with those in AD-related nonmodifiable risk factors. This finding further strengthens the tissue specificity of miRNAs. The most common is that miR-155 is involved in AD via inhibiting multiple targets and those targets might be downstream of other miRNAs which are also related to the pathological mechanism of AD. Interestingly, miR-155 can suppress the same target (e.g., SOCS1) in the different AD-related risk factors or human body systems and then jointly promote the progression of disease (Fig. 4). Such phenomenon was mainly attributed to the complexity of disease networks and the undefined crosstalk mechanism. Given those controversial results, it is necessary to make more efforts to clarify its direct targeted effects and stage-specific effects during the progression of AD.

**Table 1:** The role of miR-155 in Alzheimer’s disease: evidence from non-modifiable and modifiable risk factors

Model	Target gene	Molecular mechanisms	Outcomes	References
Aβ_1–42_ oligomers-treated HT-22 cells	N/A	N/A	Serve as an AD detector	[22]
APP/PS1 mice, Aβ1-42-treated N2a cells	SKP2	SKP2/IKKβ pathway	Aggravating Aβ deposition	[45]
Intravitreal injection of Aβ oligomers	N/A	TGF-β and prion diseases pathways	Regulating Aβ-induced retinal damage	[49]
3xTg-AD mice	SOCS-1	TNFSF10/miRNA-155/SOCS-1 network	Downregulation of the Aβ deposits and phosphorylated Tau (p-Tau)	[50]
APPtg and TAUtg mice	MSK1, GSK3β	Repressing the expression of GSK3β	Regulating amyloid-beta production or TAU phosphorylation	[51]
hTau mice	N/A	mTOR, MAPK, Wnt, and T-cell receptor signaling pathways	Phosphorylating tau	[30]
DS-like neuronal deficits in Snx27 − / − mice	C/EBPβ	miR-155/C/EBPβ/SNX27 pathway	Inducing synaptic dysfunction	[60]
3xTg-AD mice, Aβ oligomers or Aβ fibrils treated N9 microglia cells or primary astrocyte cells	SOCS1	miR-155/SOCS1/IL-6 pathway	Enhancing the expression of pro inflammatory factors	[74]
LPS-stimulated murine primary microglia	SOCS1, HIF1α, CEBPβ	TLR signal, NF-κB/RelA and STAT1/STAT3 signaling pathways	Inducing the M1-skewing of microglia	[75]
Fibrillar Aβ1-42-treated primary microglia	N/A	Regulating the endolysosomal pathway	Reducing the ability of microglia to catabolize fAβ1-42	[76]
Intraperitoneal injection of LPS	SHIP-1	miR-155/SHIP-1/p-AKT axis	Downregulation the expression of inflammatory factors	[77]
A rat model of AD	N/A	Increasing the expression of IL-1β, IL-6 and TNF-α	Aggravating neuroinflammation	[79]
Mice lacking mineralocorticoid receptors in smooth muscle cells	Cav1.2, AgtR1	miR-155/Cav1.2/AgtR1 axis	Regulating vasoconstriction in the aging vasculature	[89]
A pregnant hypertension rat model	FOXO3a	miR-155/ FOXO3a	Regulating placental tissue morphology, blood pressure and serum creatine level 30,402,830	[92]
A rat hypertension model	P27	miR-155/P27	Regulating Vascular smooth muscle cell proliferation 30468491	[94]
Two-kidney-one-clip -induced renovascular endothelial dysfunction	eNOS	NF-κB/miR-155-5p/eNOS/NO/IκB	Improving vascular endothelial function	[93]
Atherosclerotic ApoE − / − mouse model	NoxA1	miR-155-NoxA1-p47phox complex signaling pathway	Promoting vascular smooth muscle cells proliferation and neointima formation	[104]
Mir155 − / − Ldlr − / − mice and Mir155 − / − Apoe − / − mice	MafB	miR-155/ MafB/IL-6	Limiting the progression of obesity and atherosclerosis	[99]
ApoE − / − mice	NLRP3	ERK1/2/NF-κB/NLRP3 pathway	Aggravating inflammation and promoting the development of atherosclerosis	[105]
ApoE − / − mice	SOCS1	SOCS1/STAT3/PDCD4 pathway	Regulating inflammation	[106]
Controlled cortical impact-induced experimental TBI	SOCS1	SOCS1 rather than SHIP-1 pathway	Promoting post-traumatic neuroinflammatory responses	[110]
Controlled cortical impact-induced experimental TBI	N/A	Promotes the type 1 IFN response	Regulating TBI pathophysiology	[111]
Controlled cortical impact-induced experimental TBI	PGC-1α	MiR-155/PGC-1α	Suppressing mitochondrial biogenesis	[112]
Distal middle cerebral artery occlusion (dMCAO) model	Rheb	Rheb/p-AKT/ZO-1 pathway	Destroying the integrity of BBB	[115]
Middle cerebral artery occlusion (MCAO) model	TLR4	TLR4/MyD88 pathway	Resulting in the development of cell damage	[116]
Middle cerebral artery occlusion/reperfusion (MCAO/R) model	DUSP14	NF-κB and MAPKs signaling pathways	Causing cell injury and inflammation	[117]
Middle cerebral artery occlusion (MCAO) model	Rheb	Rheb/mTOR pathway	Resulting in significant cerebral infarct volumes and cell apoptosis	[118]
Middle cerebral artery occlusion/reperfusion (MCAO/R) model	MafB	miR-155/ MafB	Improving the neurological function and inhibiting inflammation response	[119]
The blood samples of major depression disorder patients, human neural progenitor cells	SIRT1	MiR-155/ SIRT1 pathway	Mediating depression and anxiety-like behaviors	[124]
Chronic unpredictable mild stress-induced depression model	N/A	Wnt/β-catenin signaling pathway	Promoting the release of inflammatory factors and the apoptosis of hippocampal neurons	[129]
Chronic unpredictable mild stress-induced depression model	FGF2	NF-κB/miR-155/FGF2	Aggravating depression and anxiety-like behaviors	[130]
MicroRNA-155 knockout mouse model and glioma mouse model	FoxO3a	FoxO3a/Akt/Stat5 signaling pathway	Repressing the proliferative and invasive abilities of CD8 + T cells	[145]
Immunological diseases-related models	SOCS1, SHIP1, c-Fos, SIRT1, c-Maf, FOXO3 etc	SOCS1/Stat5, SHIP1/AKT, SIRT1Foxop3 signaling pathway	Regulating CD4 + T cells	[147]

## Data Availability

The data that support the findings of this study are available from the corresponding author upon reasonable request.
